# Test Case Prioritization, Selection, and Reduction Using Improved Quantum-Behaved Particle Swarm Optimization

**DOI:** 10.3390/s22124374

**Published:** 2022-06-09

**Authors:** Anu Bajaj, Ajith Abraham, Saroj Ratnoo, Lubna Abdelkareim Gabralla

**Affiliations:** 1 Machine Intelligence Research Labs (MIR Labs), Auburn, WA 98071, USA; ajith.abraham@ieee.org; 2 Department of Computer Science and Engineering, Guru Jambheshwar University of Science and Technology, Hisar 125001, India; ratnoo.saroj@gmail.com; 3 Department of Computer Science and Information Technology, College of Applied, Princess Nourah Bint Abdulrahman University, P.O. Box 84428, Riyadh 11671, Saudi Arabia; lagabralla@pnu.edu.sa

**Keywords:** regression testing, nature-inspired algorithms, test case prioritization, test case reduction, test case selection, particle swarm optimization, QPSO

## Abstract

The emerging areas of IoT and sensor networks bring lots of software applications on a daily basis. To keep up with the ever-changing expectations of clients and the competitive market, the software must be updated. The changes may cause unintended consequences, necessitating retesting, i.e., regression testing, before being released. The efficiency and efficacy of regression testing techniques can be improved with the use of optimization approaches. This paper proposes an improved quantum-behaved particle swarm optimization approach for regression testing. The algorithm is improved by employing a fix-up mechanism to perform perturbation for the combinatorial TCP problem. Second, the dynamic contraction-expansion coefficient is used to accelerate the convergence. It is followed by an adaptive test case selection strategy to choose the modification-revealing test cases. Finally, the superfluous test cases are removed. Furthermore, the algorithm’s robustness is analyzed for fault as well as statement coverage. The empirical results reveal that the proposed algorithm performs better than the Genetic Algorithm, Bat Algorithm, Grey Wolf Optimization, Particle Swarm Optimization and its variants for prioritizing test cases. The findings show that inclusivity, test selection percentage and cost reduction percentages are higher in the case of fault coverage compared to statement coverage but at the cost of high fault detection loss (approx. 7%) at the test case reduction stage.

## 1. Introduction

With the advent of healthcare applications and the tremendous amount of information processing, there is a need for fault handling [1]. Therefore, software testing is becoming essential for critical safety systems, e.g., IoT devices and sensor networks connected with it in one or another, where failure may lead to loss of money and life. In other words, it is an important part of the software development lifecycle since it ensures that the software is of high quality. It accounts for around half of the entire cost [2]. Testing during the evolution and maintenance phases becomes more important to assure the software’s dependability. All of the test cases must be re-implemented to guarantee that the quality is not affected; this is known as regression testing [3]. In other words, the software is continually changing to sustain the competitive market by updating and maintaining to satisfy the changing needs. Complete retesting accounts for around eighty percent of the entire maintenance cost [4]. On the other hand, it is difficult to test each upgraded version of software nowadays. Software becomes more complex with frequent upgrades, and the amount of time and effort required for regression testing may increase. Test case reduction, selection and priority strategies can help solve these bottleneck problems [5].

1. Test Case Prioritization (TCP)
  It ranks the test cases based on some predefined goals, such as maximum code coverage, fault coverage and requirements coverage. It finds $T_{i}\in PT$ such that $\left(\forall T_{j}\right)\;\left(T_{j}\in PT\right)\;\left(T_{j}\neq T_{i}\right)\;\left[f\left(T_{i}\right)\geq f\left(T_{j}\right)\right]$; for a given test suite, *T*, its permutations set PT, and *f* denotes a function from PT to real numbers [2]. In other words, it claims to identify $T_{i}$ from PT with a value of $f(T_{i})$ larger than any other test case $\left(T_{j}\right)$ in PT. The coverage rate is represented by *f*, which calculates the performance of the permutation series in terms of real numbers.
2. Test Case Selection (TCS)
  It chooses essential test cases that are linked with the update of the software. In other words, it finds a subset of *T*, $T_{i}$ for testing the modified version of *P*, $P_{i}$ [5].
3. Test Case Reduction (TCR)
  It focuses on removing redundant test cases by finding a representative test case set, $T_{i}$, from *T* that satisfies test requirements set *R*: {r1,⋯,rn} for the defined coverage of the program [2].

TCP is the most commonly used out of these three strategies by researchers because it does not eliminate or pick test cases. Instead, it simply rearranges them such that the most critical ones are checked first. The significance of these test cases is determined by a number of factors. It might be code coverage, fault coverage, requirement priority or critical components [3]. TCS and TCR, on the other hand, may leave out certain crucial test cases that can be useful for upcoming versions of the product [2]. On the other side, finding the best order for the test cases, as well as the best way to limit or choose the test cases, makes it a NP- hard problem [6].

Optimization strategies can be used to successfully overcome these issues. Nature-inspired algorithms have been successfully employed to solve difficult optimization problems in many domains [7]. Alternatively, they can improve the cost-effectiveness of regression testing. Nature-inspired algorithms appeal to researchers because of their basic structure and ease of use. The methods are theoretically built by modeling natural events [3]. These algorithms are broadly classified into three classes: biology-inspired, physics/chemistry-inspired and social-phenomena-inspired algorithms. These techniques have also been applied in regression testing [2]. The most often used algorithms are evolutionary algorithms and swarm intelligence-based algorithms from the biology-inspired family of nature-inspired approaches [8].

PSO algorithms have been used by researchers for solving regression testing problems. We have also used similar approaches in our previous works. For Example, Dragonfly was hybridized with PSO for prioritizing the test cases using fault coverage information. It reduced the test suite to 87–96%, which thereby removed some of the critical test cases. Therefore, tri-level regression testing was performed by layering the test case selection in between the test case prioritization and reduction. The promising results of the nature-inspired algorithms on statement coverage motivated us to validate the results on fault coverage as well. As a result, this work analyses the effect of fault and statement coverage criteria on the performance of the technique. It also suggests a swarm-intelligence-based algorithm, Quantum-behaved particle swarm optimization (QPSO) and its improved version, IQPSO, for tri-level regression testing to improve the quality of results. The main contributions of this research are:

- Improved QPSO Algorithm to solve the TCP for fault and statement coverage criteria.
- Extended the algorithm for selecting the modification-revealing test cases using historical information and further reduction of the test suite size.
- Performance analysis of the algorithms using different testing goals, i.e., code coverage and fault coverage.
- Verified robustness of the proposed algorithm against Genetic Algorithm (GA), Bat Algorithm (BAT), Grey Wolf Optimization (GWO), Particle Swarm Optimization (PSO), Adaptive PSO (AdPSO) and the hybrid of PSO with Gravitational Search Algorithm (PSOGSA) and Dragonfly Algorithm (DAPSO).

Alternatively, the nature-inspired algorithms prioritize test cases based on the most extensively used criteria: statement and fault coverage. The modification-revealing test cases are included with the help of the adaptive test case selection method. It takes into account test case history and picks failed test cases based on probabilistic potentials. Since the test selection percentage is large at the expense of high inclusiveness; the TCR is introduced to minimize test suite size by removing duplicate test cases. The empirical results show that the proposed technique works for both fault and statement coverage. Inclusivity, test selection percentage and cost reduction percentages are higher in the case of fault coverage compared to statement coverage but at the cost of slightly high fault detection loss at the test case reduction stage.

The organization of the paper is structured as follows: Section 2 describes the research work done in the application of PSO algorithms for solving regression testing problems. In succession, Section 3 presents the working mechanism of the basic PSO and QPSO. The proposed algorithms are discussed in Section 4. Section 5 and Section 6 present the experimental setup and results analysis. The paper is concluded in Section 7.

## 2. Literature Review

A review of the literature on the applications of nature-inspired algorithms for regression testing is presented in this section. For Example, Li et al. [4] compared search-based methods to traditional algorithms. It was discovered that the search space is better explored with GA. It prompted more research into the use of nature-inspired algorithms. For example, Zhang et al. [9] used a distance-based and index-based implementation of ACO to prioritize test cases, and the results were superior to GA, PSO and RS. Using the Cuckoo Search Algorithm (CSA), a new fixup mechanism for permutation encoding was developed to address the TCP problem [3]. CSA was also used to reduce the test suite for configuration-aware software testing [10].

Mohapatra and Prasad [11] have employed Ant Colony Optimization on Java programs, and analyzed the performance for reduced suite and complexity to traditional techniques. The quantum-inspired ACO approach for test suite reduction was developed by Zhang et al. [12]. The suggested method outperformed previous ACOs in terms of a % decrease in size. NSGA-II was employed by Mondal et al. [13] for TCS by taking the test suite variety and code coverage as a fitness metric. With a maximum time limit of 20%, it was discovered that diversity enhanced the defect detection rate by up to 16 percent.

Several researchers have employed PSO, such as Khatibsyarbini et al. [14], who used string distances to arrange the test instances and validated it on the real-world TSL dataset. To choose test cases based on redundancy, binary constraint PSO and its hybrid variants with local search algorithms were developed [15]. PSO was implemented with local search to select test cases, having goals of increased branch coverage and lower costs [16]. Because of the positive findings, PSO was also combined with harmony search, which performed better than NSGA-II [17]. Correia [18] developed a test suite diversity diagnosability measure, and the results were improved by applying local search algorithms with PSO to maximize requirement coverage while lowering associated costs.

The test case reduction was also implemented with TCP by hybridizing the PSO with the Dragonfly Algorithm. The observations suggested that hybrid algorithms outperformed other search algorithms [6]. Tri-level regression tesing was proposed to prioritize, select and minimize the test cases based on statement coverage. It was observed that the hybrid of PSO with Gravitational Search Algorithm (PSOGSA) outperformed GA, PSO and GSA [5]. The test suite was minimized using hybrid PSO and the Firefly Algorithm, considering the fault coverage [19]. The modified condition decision coverage criteria were employed as fitness measures in PSO for prioritizing the test cases [20]. Deneke et al. [21] also proposed the PSO Algorithm for reducing the test suite based on requirement coverage and cost. Samad et al. [22] proposed multi-objective PSO for optimizing the code and fault coverage and cost. Agrawal and Kaur selected the test cases using the fault information with the application of PSO [23]. Table 1 shows the application of PSO Algorithms for regression testing methods along with their optimization criteria.

PSO algorithms have become one of the state-of-the-art algorithms and show promising results in various domains, e.g., reduction of CO${}_{2}$ emissions in air baggage systems [24]. The original PSO, on the other hand, had issues, such as getting trapped in local optima and premature convergence [25]. According to the findings, upgraded and hybrid versions of PSO outperformed PSO for complicated systems [15]. One such algorithm is Quantum-behaved PSO (QPSO). It is based on quantum mechanics in which particles can travel through a large search space for global convergence [26]. This method has shown good results in a variety of applications, such as cancer classification [27], feature extraction ([28,29,30]) and constrained engineering problems [31], and others [32]. However, it has not been investigated in the TCP domain, which might be due to the fact that it was originally designed for continuous problems. Therefore, to shift infeasible solutions into feasible ones, we suggested a discrete QPSO method based on an adaptation strategy. It does, however, have significant drawbacks, such as early convergence. As a result, we have improved it with a dynamic contraction-expansion coefficient to speed up the performance in the last iterations [31].

Besides this, we have extended the algorithm for selecting the modification-revealing (MR) test cases from the current best solution of TCP. It is occasionally necessary to reduce the test suite by reducing redundancy because of time limits; thus we employed the TCR approach in the end. Our key focus in this research is on performing regression testing in three steps, including TCP, TCS and TCR processes for fault and statement coverage. Alternatively, the effect of different testing criteria on the overall performance of algorithms was analyzed. The observations suggest that the tri-level regression technique is effective for both coverage criteria. The proposed algorithm, IQPSO, is statistically not significant from PSOGSA; however, its variance and mean fitness values are better.

## 3. Preliminaries

This Section briefly explains the working mechanism of Particle Swarm Optimization (PSO) and Quantum-behaved PSO.

### 3.1. Particle Swarm Optimization

PSO is inspired by particle behavior, such as flocking, swarming and herding. Each particle changes its flight based on self or companion’s previous flight experience. Each particle, based on its own experience, is aware of the location of food, which is referred to as the personal best position (*P*). Simultaneously, the particle has knowledge of the swarm’s best-discovered position, the global best position (*G*). This phenomenon is reproduced in order to solve real-world issues. In other words, the swarm is made up of particles that fly randomly in the solution space with velocity $v_{i}$ at position $x_{i}$ and change positions based on personal experience, social behavior and cognitive behavior ([33]). The position and velocity of each particle *i* at tth generation are defined mathematically as:

(1) $$v_{i}\left(t+1\right)=wv_{i}+c_{1}r_{1}\left(P_{i}\left(t\right)-x_{i}\left(t\right)\right)+c_{2}r_{2}\left(G\left(t\right)-x_{i}\left(t\right)\right)$$

(2) $$x_{i}\left(t+1\right)=x_{i}\left(t\right)+v_{i}\left(t+1\right)$$

*w* is the inertia weight used to regulate the impact of prior velocity; $c_{1}$ and $c_{2}$ are the constants used to adjust the attractiveness speeds among these social and cognitive elements; and $r_{1}$ and $r_{2}$ are uniform random values in the range [0,1].

### 3.2. Quantum-Behaved PSO

A more robust variant of PSO called QPSO is created [25], as PSO cannot ensure global convergence [32]. It determines the Quantum-behaved particles’ route, assuming that *N* particles with specified energy and delta potential are well-centered in each dimension of the n-dimensional Hilbert search space. The jth component of tje particle’s position at tth iteration is given by the Monte Carlo technique:

(3) $$x_{ij}\left(t+1\right)=a_{ij}\left(t\right)\pm \frac{L_{ij}\left(t\right)}{2}\ln\left(\frac{1}{u_{ij}\left(t\right)}\right)$$

(4) $$L_{ij}\left(t\right)=2\theta \left|mean_{j}\left(t\right)-x_{ij}\left(t\right)\right|\;and\;mean_{j}\left(t\right)=\frac{1}{N}\sum_{i}^{N}x_{ij}\left(t\right)$$

where $u_{ij}\left(t\right)$ is a uniformly distributed value between 0 and 1, $a_{ij}\left(t\right)$ is the individual’s local attractor and theta is the contraction-expansion coefficient. As a result, the particle’s location in the QPSO Algorithm may be calculated as follows:

(5) $$x_{i}\left(t+1\right)=\{\begin{array}{cc} a_{ij}\left(t\right)-\theta \left|{Mbest}_{j}\left(t\right)-x_{ij}\left(t\right)\right|\ln\left(1/u_{ij}\left(t\right)\right) & :r(0,1)>0.5\\ a_{ij}\left(t\right)+\theta \left|{Mbest}_{j}\left(t\right)-x_{ij}\left(t\right)\right|\ln\left(1/u_{ij}\left(t\right)\right) & :otherwise \end{array}\;$$

In each generation, the particles travel around the local attractor $a_{ij}\left(t\right)$, which is formed with the *P* and *G* optimal locations as follows:

(6) $$a_{ij}\left(t\right)=\phi_{ij}\left(t\right)P_{ij}\left(t\right)+\left(1-\phi_{ij}\left(t\right)\right)G_{j}\left(t\right),\;\phi_{ij}\left(t\right)∼\left(0,1\right)$$

The next generation’s particle position distribution is computed using the mean Mbest of the *P* best locations of the particles.

(7) $$Mbest_{j}\left(t\right)=\frac{1}{N}\sum_{i}^{N}P_{ij}\left(t\right)$$

The fundamental difference between the PSO and the QPSO is twofold: (1) a large search space owing to the exponential distribution of the particles; and (2) the particle’s distance from its partners is considered, whereas in PSO, particles move freely to converge to the global best. Another benefit is that it only has one parameter, theta, which must be managed for convergence and whose value is reduced linearly using the equation:

(8) $$\theta =\left(\theta_{max}-\theta_{min}\right)*\left(Maxit-t\right)/Maxit+\theta_{min}$$

Because it is simple to use and has been tried and tested on a variety of applications, ref. [32], we sought to apply the QPSO method to a discrete optimization problem in this study and compare its performance to that of state-of-the-art techniques.

## 4. Proposed Work

This section explains an improved QPSO Algorithm. It is described in three stages. First, it incorporates the asexual reproduction operator into the population (ARO). Second, the adaptive contraction-expansion coefficient is used to alleviate the issue of stagnation. Finally, the adaptive TCS method is then used to choose the MR test cases. It is followed by the TCR technique for minimizing the size of the test suite as follows:

### 4.1. Population Update

Appropriate mapping increases the algorithm’s speed and efficacy, so the real numbers are being updated to permutation series by applying the asexual reproduction method. The fix-up process creates a link between real numbers and test case sequences, such that the current solution acquires the parent solution’s properties by forming the bud from the parent while keeping the offspring’s possible values (larva). Alternatively, the algorithm recalculates and rounds the output to natural values. Do not care conditions (*) are used to replace out-of-range and identical particles [3].

### 4.2. Dynamic Contraction-Expansion Coefficient (θ)

The value of the contraction-expansion coefficient θ in QPSO indicates the population’s search radius. The bigger the value, the wider the particle search range; on the other hand, the smaller value, the more narrow the search range. The evolution velocity coefficient α is introduced to adaptively alter θ [34]:

(9) $$\alpha =\frac{Gfit(t)}{Gfit(t-1)}$$

α∈(0,1) since the global optimal solution is always replaced by the solution with superior fitness as the iteration advances; specifically, Gfit(t)>>Gfit(t−1)>0. The fitness value of global best varies significantly when the value of α is tiny. The evolution is speeding up at small α due to significant changes in Gfit as the particles are beyond the ideal location. As a result, θ needs to be increased to ensure a quick optimization. The evolution gets slowed down with large α, and the particle search range is reduced, and so is the θ for better optimization. The solution converges, and the evolution stops at α=1. Therefore, Equation (8) is replaced with:

(10) $$\theta =\theta_{max}-\alpha \theta_{min}$$

here $\theta_{min}$ and $\theta_{max}$ are the minimum and maximum values, and α is the evolution velocity coefficient’s weight. Further, the difference between P(t)−G(t) approaches zero as the iterations proceed, so this value is replaced with the mutation operator $x_{rj}\left(t\right)-x_{sj}\left(t\right)$, where *r* and *s* are the particles selected randomly from the population [34]. It is mathematically formulated as:

(11) $$x_{ij}\left(t\right)=\phi_{ij}\left(t\right)\left(x_{rj}\left(t\right)-x_{sj}\left(t\right)\right)+\left(1-\phi_{ij}\left(t\right)\right)G_{j}\left(t\right)\pm \theta \left|{Mbest}_{j}\left(t\right)-x_{ij}\left(t\right)\right|\ln\left(1/u_{ij}\left(t\right)\right)$$

### 4.3. Adaptive Test Case Selection

The test case selection approach was proposed [5] that takes into account the statements they cover in the modified version and the impact of failed test cases. It is a dynamic algorithm that picks test cases depending on their pass or fail information after each iteration of TCP step. During the selection process, it requires exact input information, which is quite important in uncovering errors. It is called adaptive as it revises the fault detection capabilities (P(t)) of unallocated test cases and chooses test cases based on existing and earlier historical data. The technique is described as:

(12) $$Pot\left(s\right)=\{\begin{array}{cc} Pot^{\prime }\left(s\right) & :s\;is\;not\;run\;by\;t^{\prime }\\ Pot^{\prime }\left(s\right)*q & :s\;is\;run\;by\;t^{\prime }\;and\;t^{\prime }\;is\;failed\\ Pot^{\prime }\left(s\right)*p & :s\;is\;run\;by\;t^{\prime }\;and\;t^{\prime }\;is\;passed \end{array}\;$$

Before picking the test case $t^{\prime }$, Pot(s) is the chance of any statement *s* having additional errors. *p* and *q* are constants with values between 0 and 1, such that p+q=1. It measures the influence of pass or fail status on the Pot(s) of any statement *s*. The values of *p* and *q* are set to 0.15 and 0.85, and *q* is set high since the goal is to acquire a larger proportion of failed test cases than passed ones. It also uses Equation (13) to update the P(t) of the unallocated test cases *t*:

(13) $$P\left(t\right)=\sum_{s\;is\;run\;by\;t}Pot\left(s\right)$$

The test case *t* with the highest P(t) is chosen, and other test cases’ potentials are updated using the chosen test case’s state. The P(t) of unassigned test cases *t* is also updated depending on their revised potentials. In other words, the unassigned test cases’ fault detection capacity is recalculated. The shortlisting sequence is based on the most recent data obtained, and it picks the test case *t* that has the highest rank in the modified P(t). If the test cases are tied, the initial copy of the test case order is used to break the tie. ST is created by removing the specified test case from PT. The previous steps are continued until the stopping requirements are fulfilled, i.e., it creates a sufficient test case to achieve 100 percent statement and fault coverage (mx), as presented in Algorithm 1. As the algorithm contains two loops that run up to the size of the test suite, the time complexity of the algorithm is $O(n^{2})$.
**Algorithm 1** Adaptive Test Case Selection (ATCS) Algorithm.1:Define potentials p and q, p + q = 12:Initialize PT = Prioritized suite ST = empty test set with capacity mx3:Select the first *r* test cases that covers all faults and statements4:st = 1 and t = PT(1) = ST(1)5:Find Pot(s) and empty PT(1)6:*Find other test cases needed for full coverage*7:**while** size(PT)>0 **do**8:      **if** (st = mx) **then**9:            break10:     **end if**11:     st = st + 112:     **for** t **do** = 1:PT Calculate P(t)13:     **end for**14:     t = max(P)15:     Update Pot(s), PT = PT − t, ST = ST + t16:     Empty P array for reassignment17:**end while**18:Return selected test cases

### 4.4. Test Case Reduction (TCR)

To reduce suite size and cost, the current best solution of every generation is followed by the duplicate verification, and the very first RT test cases covering the faults/statements completely are chosen. This technique has the advantage of exposing how the test cases are ranked precisely [6]. The pseudo-code of TCR is given in Algorithm 2. Since it contains one loop for the test cases (n) and another for finding the faults or statements (m), so the time complexity of the algorithm can be calculated as O(nm).

Algorithm 3 presents an Improved IQPSO Algorithm consisting of two loops, Maxit and Pop. It also contains ATCS and TCR algorithms, so the overall complexity of the algorithm is $O(Maxit*Pop*\left(n^{2}+nm\right))$.
**Algorithm 2** Test Case Reduction Algorithm.1:Define test fault matrix M, Ranked test cases T,2:Initialize Faults position array Pos and test cases indices array I3:**for** t=1,2,…,size(T) **do**4:      **for** f=1,2,…,size(Pos) **do**5:             **if** (M(T(t), Pos(f)) = 1 and I(f) = 0)) **then**6:                   I(f) = t7:             **end if**8:      **end for**9:**end for**10:Reduced array R = T(I)

**Algorithm 3** IQPSO Algorithm.
1:Define Pop, Maxit, $\theta_{max}$, $\theta_{min}$ and δ2:Initialize random population $x_{i}$3:**for** t=1,2,…,Maxit **do**4:      **for** i=1,2,…,Pop **do**5:             Calculate fitness $f(x_{i})$6:             Update $P_{i}$ and *G* solutions7:             Update alpha and theta using (9) and (10)8:             **if** α==1 for δ attempts9:             Update $x_{i}\left(t+1\right)$ using (11)10:           **end if**11:           Update $x_{i}\left(t+1\right)$ using (5)12:     **end for**13:     Apply ATCS Algorithm 114:     Apply TCR Algorithm 215:
**end for**
16:Return: Final solution


## 5. Experimental Setup

This section outlines the empirical study, including research questions, datasets, evaluation metrics and the algorithms with which the proposed algorithm is compared. The formulated research questions are:

**RQ1. What is the performance of the proposed algorithm for TCP?**

The objective is to see if the suggested algorithm outperforms others. It also identifies which algorithm produces the best results, as well as the effect of various testing settings on algorithm performance.

**RQ2. What is the performance of the proposed algorithm for TCS?**

The goal is to investigate the efficiency of the provided strategies for the ATCS method, namely, test selection percentage, inclusivity of MR test cases and reduction in cost percentage.

**RQ3. What is the performance of the proposed algorithm for TCR?**

The aim is to evaluate the effectiveness of the suggested algorithm to that of the other methods. Furthermore, to figure out which testing criteria improve TCR. Alternatively, to see how it impacts the coverage and fault detection capabilities of the test suite.

### 5.1. Experimental Design

PSO, QPSO and the latest variants of PSO, i.e., PSOGSA [5], DAPSO [6] and Adaptive PSO (AdPSO) [35], are the algorithms considered for comparison. Apart from these, the algorithm is also validated against state-of-the-art algorithms, such as GA, BAT and the recently proposed Grey Wolf Optimization (GWO) [36]. These methods were developed using MATLAB R2017 on a Dell laptop with an Intel i5 CPU, Windows 11 and 8GB of RAM. Due to their stochastic nature, the algorithms are performed 30 times. These are used on three different Java applications (jtopas, ant and jmeter) that are pulled from the software infrastructure repository (SIR) [37]. We have applied the algorithm to different versions of these programs. Table 2 provides more information.

The performance of the algorithms is influenced by parameter choices [38]. As a result, we carefully choose the parameters based on a comprehensive review of related works as well as a trial-and-error process for determining optimal values. Table 3 also contains the data retrieved using the Taguchi approach.

### 5.2. Performance Measures

The following performance measures were used to validate the efficiency and efficacy of these algorithms:

#### 5.2.1. Test Case Prioritization

To assess the robustness of the proposed technique, the test cases were selected using two separate testing criteria: fault and statement coverage. As a result, the commonly used fitness measurements and effectiveness measures are defined as follows:

*Average Percentage of Fault Detection (APFD)* is a measure of how well a system detects faults. It finds a weighted average of the detected defects based on where they are in the test suite [39]. It is computed as follows:

(14) $$APFD=1-\frac{\sum_{i=1}^{m}TF\left(i\right)}{n*m}+\frac{1}{2*n}$$

The location of the test case that detects the ith fault is denoted by TF(i), and the faults covered by *n* test cases is denoted by *m*. Its value lies between 0 and 100, with greater being better. The Average Percentage of Statement Coverage (APSC) is calculated in the same way as the APFD.

#### 5.2.2. Test Case Selection and Reduction

Test selection percentage, cost reduction percentage, fault detection percentage and coverage loss percentage are commonly used efficacy measures. In addition to these, the inclusivity measure is also used for TCS as follows:

*Test Selection Percentage (TSP)*: It is a percentage selection in the size of the test suite.

(15) $$TSP=\frac{st}{n}*100$$

here st indicates the test cases selected from *n* test cases.

*Inclusivity (I)*: The extracted MR test cases emr divided by the total MR test cases totmr gives the inclusivity measure.

(16) $$I=\frac{emr}{totmr}*100$$

*Fault Detection Loss Percentage (FDLP)*: The ratio of faults not covered by minimized test suite nfl to total faults covered by the original suite tfc [40]:

(17) $$FDLP=\frac{nfl}{tfc}*100$$

*Cost Reduction Percentage (CRP)*: It is a percentage of the test suite’s cost that is reduced rcost when compared to the original suite’s cost tcost.

(18) $$CRP=\frac{rcost}{tcost}*100$$

## 6. Results and Analysis

This section experimentally assesses the proposed algorithm for TCP using statement and fault coverage criteria. TCS and TCR have been studied for their effects on fault coverage loss, statement coverage loss and cost benefits. To determine the experimental results of a software, the cumulative average of all its iterations was employed. The performance metrics for each version were calculated using the average of 30 runs. For fitness metrics, boxplots and convergence curves are also shown. A one-way ANOVA test with a *p*-value =0.05 was used to analyze the algorithms’ output statistically. If p<0.05, the null hypothesis was rejected, suggesting that the algorithms’ difference was statistically significant. Further, Tukey’s simultaneous test was used to evaluate the pair-wise comparison of the methods.

### 6.1. Performance Analysis of TCP (RQ 1)

**Figure 1:**
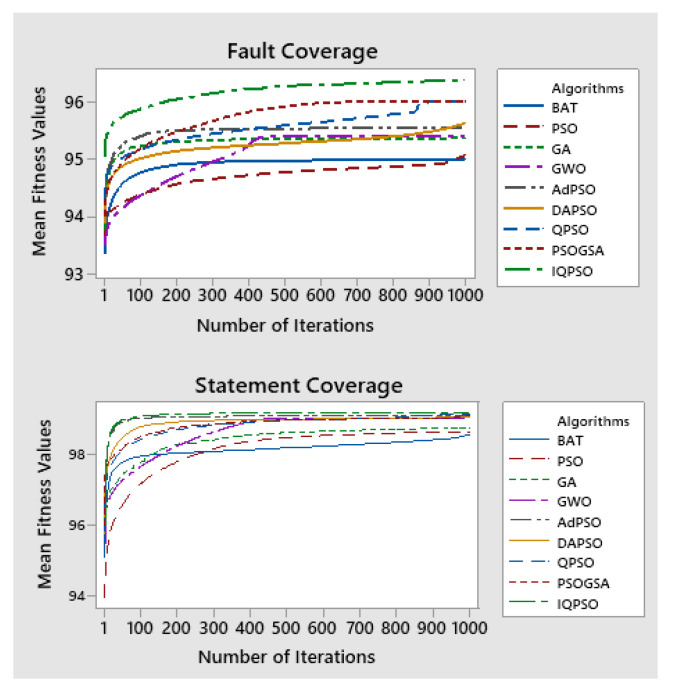
Convergence curves of algorithms for fault and statement coverage criteria of TCP.

Table 4 shows the mean fitness and variance of performance metrics as well as their corresponding Tukey group ranks for all of the programs. The observations state that IQPSO is statistically different from all other nature-inspired algorithms, with a *p*-value of less than 0.05, for both statement and fault criteria except PSOGSA. Moreover, it suggests that there is no significant difference between the means of (1) PSOGSA and QPSO, (2) AdPSO and DAPSO, (3) AdPSO and GWO and (4) GA and PSO for both criteria. The convergence curves for one of the versions of the subject programs are illustrated in Figure 1. It shows that the proposed IQPSO Algorithm possesses high-quality solutions for both criteria.

**Figure 2:**
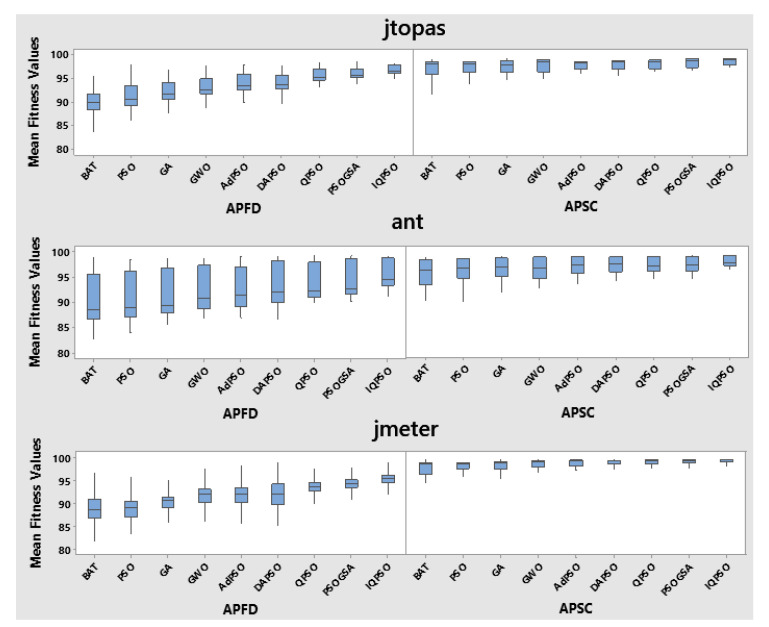
Boxplots of algorithms for fault and statement coverages of TCP.

It was also observed that most of the algorithms had equivalent variance in the case of statement coverage. The boxplots in Figure 2 also depict variation in algorithmic performance for fault coverage that is higher for statement coverage. This is because faults are dispersed across the entire software. In other words, most test cases cover almost the same statements. Therefore, the statement coverage boxplots are more compressed than the fault coverage boxplots. Overall, the proposed IQPSO is superior to all other algorithms in terms of variance.

### 6.2. Performance Analysis of TCS (RQ 2)

The performance of the test case selection is evaluated using test selection percentage, inclusivity and cost reduction percentages as follows:

#### 6.2.1. Test Selection Percentage (TSP)

The full version study revealed a random pattern, indicating that all of the algorithms behave similarly. We are unable to determine which algorithm is superior to the others. However, according to the program analysis, DAPSO produced the best TSP for two out of three programs in both coverages (see Table 5). It can be observed that IQPSO, PSOGSA, QPSO and AdPSO are better than PSO, GWO, GA and BAT for fault coverage. On the other side, GA, GWO and PSO performed better than IQPSO, PSOGSA, AdPSO, QPSO and BAT in the case of statement coverage. IQPSO performed better for *ant*, which includes a significant number of test cases and statements. As a result, it can be said that the improved approach may outperform the large programs. It was also observed that the selection percentage is less in the case of fault coverage than the statement coverage.

#### 6.2.2. Inclusivity (I)

All the algorithms are capable of incorporating over 78% and 76% MR test cases in statement and fault coverages. The proposed algorithm worked well for statement coverage, followed by PSOGSA, QPSO, DAPSO, AdPSO, PSO, GWO, GA and BAT. However, IQPSO and PSOGSA performed least well in the case of fault coverage and the performance-wise algorithms can be ranked as AdPSO, BAT, PSO, GA, QPSO, DAPSO, GWO, IQPSO and PSOGSA. Alternatively, the ATCS method picks a large number of test instances in the case of statement coverage than the fault coverage criteria. Table 5 also showed that the fault coverage criteria is better for inclusiveness of the MR test cases. The inclusivity of variable state test cases is critical since they necessitate extra care because they do not produce the same results for all versions. In other words, the ATCS method is based on the modification coverage so the fault coverage is the more appropriate choice for inclusivity of the MR test cases over statement coverage.

#### 6.2.3. Cost Reduction Percentage (CRP)

TCS has witnessed a cost reduction of 6.93–30.26% and 18.78–48.73% for statement and fault coverage criteria. In the case of *ant* and *jmeter*, DAPSO delivers the best cost reduction % in most of the cases, whereas IQPSO outperformed DAPSO for statement coverage in *ant* and fault coverage in *jtopas*. In other words, DAPSO, GA, GWO and PSO performed better than the PSO variants in statement coverage, whereas IQPSO is the first runner-up for fault coverage after DAPSO (see Table 5). It was also discovered that the TSP and the CRP have an indirect link. In other words, the lower the number of tests in the suite, the higher the CRP.

### 6.3. Performance Analysis of TCR (RQ 3)

The performance of the test case reduction is analyzed by calculating the test selection percentage, cost reduction percentage and fault detection loss percentages as follows:

#### 6.3.1. Test Selection Percentage (TSP)

Table 6 shows that all the methods perform almost equally well when it comes to reducing the test suite. Nonetheless, the proposed approach performed better than other nature-inspired algorithms for both coverages. Comparatively, BAT had a higher selection percentage. TSP was larger for statement coverage than fault coverage. It is because there was a lot of redundancy in statement coverage, and the faults were spread over the whole program and to balance them APSC had slightly higher TSP than APFD.

#### 6.3.2. Fault Detection Loss Percentage (FDLP)

The incorporation of ATCS helped the TCR in reducing the suite size with complete statement coverage and minimized the fault loss too. The findings reveal that the direct application of TCR gave quite a high fault loss, i.e., between 5% and 40% [40]. AdPSO outperformed the other methods for statement (0–0.318%) as well as fault coverages (0–2.887%). Table 6 shows that IQPSO had the least loss in statement coverage compared to the other algorithms, except *jtopas*, where DAPSO worked better. The observations also depict that the fault loss in APFD (0–8.134%) was higher compared to that in APSC (0–1.121%). The reason for this is that the faults are spread over the software. Hence, the fault coverage is lost by removing certain statement redundancy. It may be deduced that the loss of coverage and the reduction in test suite size are inversely proportionate.

#### 6.3.3. Cost Reduction Percentage (CRP)

The experimental findings show that the cost reduction in fault coverage was more than the statement coverage, as it reduced the test suite better too. It was also observed that cost reduction was inversely proportionate to test selection percentage, i.e., the larger the decrease in test suite size, the lower the running cost. TCM costs were estimated to be roughly 60 and 40 percent lower than TCS for statement and fault coverage. Table 6 clearly shows that the CRP of IQPSO outperformed all other algorithms, followed by PSOGSA, QPSO and DAPSO. GA, PSO, GWO and AdPSO had nearly identical performances. Overall, IQPSO demonstrated superior search capabilities for solving the regression testing problem in all three subject programs.

## 7. Conclusions

In this paper, we have suggested an improved QPSO Algorithm for regression testing and validated it against GA, GWO, BAT and PSO and its variants, DAPSO, PSOGSA and AdPSO. The empirical results show that the proposed algorithm IQPSO has a comparatively low variance for statement and fault coverages. Further, the adaptive test selection approach was able to successfully identify 77–96% of the MR test cases in both fault and statement coverages. The study also revealed that the adaptive test selection percentage of fault coverage was 40–60% less than the statement coverage with high inclusivity. IQPSO performed better than all other algorithms for test case reduction and cost reduction %. The algorithms showed approximately a 7% difference in the fault detection capability loss for fault coverage over statement coverage. In the future, we will strive to reduce this fault detection loss to almost zero and validate the algorithm’s results in a variety of large-scale real-world applications. We intend to investigate alternative variants of QPSO Algorithms by modification and hybridization to improve the inclusivity and algorithm’s performance even further.

## Figures and Tables

**Table 1:** Summary of PSO Algorithms used in Regression Testing.

Author(s) (Year)	Method	Nature-Inspired Approaches	Criteria
De Souza et al., 2013, 2014	TCS	Binary PSO	Requirement Coverage with Time
De Souza et al., 2015	TCS	Binary PSO-HS	Branch Requirement Coverage with Cost
Khatibsyarbini et al., 2018	TCP	PSO	String Distances
Agrawal and Kaur 2018	TCS	PSO	Fault Coverage and Time
Correia, 2019	TCS	PSO-LS	Requirement Coverage
Nayak and Ray 2019	TCP	PSO	Modified Condition Decision Coverage
Samad et al., 2021	TCP	MOPSO	Code and Fault Coverage with Cost
Bajaj and Abraham, 2021	TCP and TCR	DAPSO	Fault Coverage
Bajaj and Sangwan, 2021	TCP, TCS, TCR	PSOGSA	Statement Coverage
Bharathi, 2022	TCR	PSO-FFA	Fault Coverage
Deneke et al., 2022	TCR	PSO	Requirement Coverage and cost

**Table 2:** Subject Programs.

Programs	Versions	KLOC	Classes	Methods	Test Cases	Type
ant	7	80.4	650	7524	878	JUnit
jmeter	5	43.4	389	3613	97	JUnit
jtopas	4	5.4	50	748	209	JUnit

**Table 3:** Parameter settings of the algorithms.

Algorithms	Parameter Values
GA	$p_{cr}=0.8$, $p_{m}=0.1$, tournament selection, ordered crossover
BA	$r_{o}=0.001$, $A_{o}=1$, $f_{min}=0$, $f_{max}=1.5$, α=0.9, γ=0.99
PSO, AdPSO	$c_{1}=1.5$, $c_{2}=2$, $w_{min}=0.4$, $w_{max}=0.9$
QPSO	$\theta_{min}=0.5$, $\theta_{max}=1.7$
IQPSO	$\theta_{min}=0.5$, $\theta_{max}=1.7$, δ=5
PSOGSA	c1=1.5, c2=2, $w_{min}=0.4$, $w_{max}=0.9$, α=15, $G_{0}=100$, Sinemap
DAPSO	s=0.2, a=0.25, c=0.6, f=0.8, e=0.8, c1=1.5, c2=2, $w_{min}=0.4$,
	$w_{max}=0.9$
Common Parameters	Pop=100, Maxit=1000

**Table 4:** Comparisons of the algorithms for TCP over fault and statement coverages.

Programs	Algorithms	Mean Fitness, Variance and Tukey Ranking for TCP (%)					
		APFD	Variance	TR	APSC	Variance	TR
jtopas	IQPSO	96.702	0.879	A	98.559	0.494	A
	PSOGSA	95.965	1.413	AB	98.229	0.842	AB
	QPSO	95.621	1.966	B	97.998	0.806	BC
	DAPSO	94.066	3.884	C	97.841	0.976	BCD
	AdPSO	93.93	4.576	CD	97.795	0.817	BCD
	GWO	93.118	4.299	D	97.706	1.895	DE
	GA	91.918	4.555	E	97.463	1.809	DE
	PSO	91.18	9.875	E	97.33	1.905	EF
	BAT	89.951	9.152	F	96.863	4.034	F
ant	IQPSO	95.337	7.283	A	98.105	0.976	A
	PSOGSA	94.344	10.394	AB	97.482	2.135	B
	QPSO	93.937	9.935	B	97.403	2.241	B
	DAPSO	93.292	14.327	BC	97.397	2.49	B
	AdPSO	92.53	15.674	C	97.239	2.85	B
	GWO	92.149	15.204	CD	96.586	4.991	C
	GA	91.304	17.977	DE	96.657	4.476	C
	PSO	90.577	19.975	EF	96.529	4.591	C
	BAT	90.094	20.42	F	95.924	6.397	D
jmeter	IQPSO	95.402	3.498	A	99.218	0.193	A
	PSOGSA	94.31	5.603	B	98.989	0.351	AB
	QPSO	93.696	6.492	B	98.987	0.358	AB
	DAPSO	92.137	10.134	C	98.916	0.359	B
	AdPSO	92.12	9.617	C	98.819	0.592	BC
	GWO	91.943	8.38	C	98.625	0.677	C
	GA	90.672	8.894	D	98.331	0.747	D
	PSO	89.396	10.309	E	98.22	0.703	D
	BAT	89.145	12.483	E	97.748	1.914	E

**Table 5:** Comparisons of the algorithms for TCS over fault and statement coverages.

Program Versions	Algorithms	TSP		Inclusivity		CRP	
		${TSP}_{APSC}$	${TSP}_{APFD}$	$I_{APSC}$	$I_{APFD}$	${CRP}_{APSC}$	${CRP}_{APFD}$
jtopas	IQPSO	81.235	**51.750**	**89.024**	87.834	17.356	**48.731**
	PSOGSA	81.567	53.567	88.457	86.238	18.986	48.001
	QPSO	83.485	51.833	87.422	81.034	19.084	48.767
	DAPSO	**68.960**	59	87.644	84.387	**30.265**	41.83
	AdPSO	80.518	61.5	85.087	**92.083**	19.711	29.359
	GWO	74.035	64.75	82.39	75.67	26.125	34.918
	GA	77.926	77.867	84.263	85.544	21.829	23.966
	PSO	71.593	65.5	86.672	89.792	26.897	34.695
	BAT	83.368	73.858	81.536	90.424	17.451	32.993
ant	IQPSO	**85.333**	59.667	**80.449**	79.509	**14.321**	31.527
	PSOGSA	86.468	62.879	79.298	80.687	12.686	30.878
	QPSO	88.81	67.622	79.5	89.621	10.016	40.798
	DAPSO	87.995	**55.667**	79.423	85.064	11.378	**45.799**
	AdPSO	90.905	77	78.363	**95.803**	8.487	22.966
	GWO	90.057	72.533	78.506	88.889	8.961	27.57
	GA	89.914	72.944	77.68	93.953	9.054	28.057
	PSO	91.014	63.889	79.752	94.038	7.887	36.914
	BAT	89.676	79.333	78.583	94.915	9.259	20.893
jmeter	IQPSO	86.483	62.4	**96.940**	78.305	12.518	35.895
	PSOGSA	88.567	63.588	95.876	77.365	11.568	34.757
	QPSO	91.476	66.15	94.828	84.463	6.934	31.637
	DAPSO	**82.016**	**61.540**	94.149	83.014	**15.728**	**36.501**
	AdPSO	90.843	71.89	96.02	**96.367**	7.656	26.219
	GWO	82.903	74.19	91.695	87.447	15.025	23.725
	GA	82.363	77.51	91.557	95.509	15.251	21.195
	PSO	84.343	69.64	94.413	88.404	14.75	27.25
	BAT	86.223	78.37	93.147	92.617	12.894	18.783

The best results are highlighted with bold.

**Table 6:** Comparisons of the algorithms for TSR over fault and statement coverages.

Program Versions	Algorithms	TSP		FDLP		CRP	
		${\mathrm{TSP}}_{\mathrm{APSC}}$	${\mathrm{TSP}}_{\mathrm{APFD}}$	${\mathrm{FDLP}}_{\mathrm{APSC}}$	${\mathrm{FDLP}}_{\mathrm{APFD}}$	${\mathrm{CRP}}_{\mathrm{APSC}}$	${\mathrm{CRP}}_{\mathrm{APFD}}$
jtopas	IQPSO	**22.085**	**19.642**	0.974	1.707	**77.911**	**82.373**
	PSOGSA	22.138	19.711	0.902	1.909	77.656	81.876
	QPSO	22.341	19.717	0.905	2.302	77.85	81.253
	DAPSO	22.626	19.742	**0.000**	1.372	77.576	81.333
	AdPSO	23.593	23.45	0.974	**0.000**	76.44	76.45
	GWO	22.426	20.583	0.905	1.949	77.71	80.775
	GA	23.718	21.783	1.112	**0.000**	76.569	78.67
	PSO	22.626	21.842	0.835	1.064	77.505	79.164
	BAT	24.518	24.642	0.399	0.764	75.446	75.894
ant	IQPSO	**30.998**	**27.933**	**0.318**	8.134	**69.275**	**73.407**
	PSOGSA	31.755	28.234	0.412	6.689	68.587	73
	QPSO	31.425	27.933	0.434	5.975	67.295	72.07
	DAPSO	31.283	29	0.337	**2.887**	67.288	71.014
	AdPSO	32.278	30	**0.318**	3.998	66.886	70.209
	GWO	33.267	29.667	0.89	5.888	65.408	70.127
	GA	33.891	27.780	0.359	7.982	65.028	73.513
	PSO	32.61	28.333	1.121	4.572	65.855	72.17
	BAT	35.291	30.333	0.446	3.696	64.574	70.274
jmeter	IQPSO	**21.767**	**19.000**	**0.000**	1.387	**77.132**	**81.248**
	PSOGSA	22	19.354	**0.000**	1.076	76.453	80.653
	QPSO	22.272	19.94	**0.000**	0.752	76.541	79.73
	DAPSO	22.003	20	**0.000**	2.331	76.535	78.874
	AdPSO	23.088	21.2	**0.000**	**0.000**	75.643	77.755
	GWO	22.834	22.4	**0.000**	0.321	76.349	77.455
	GA	22.684	21.47	**0.000**	**0.000**	76.193	78.171
	PSO	22.044	21.4	**0.000**	0.752	76.859	77.102
	BAT	23.75	23.16	**0.000**	0.357	74.837	77.237

The best results are highlighted with bold.

## Data Availability

Not applicable.

## Abbreviations and Nomenclature

### Abbreviations

ACO	Ant Colony Optimization
AdPSO	Adaptive Particle Swarm Optimization
ANOVA	Analysis of Variance
APFD	Average Percentage of Fault Detection
APSC	Average Percentage of Statement Coverage
ARO	Asexual Reproduction Operator
ATCS	Adaptive Test Case Selection
BAT	Bat Algorithm
CRP	Cost Reduction Percentage
CSA	Cuckoo Search Algorithm
DA	Dragonfly Algorithm
FDLP	Fault Detection Loss Percentage
FP	Fault Position Array
GA	Genetic Algorithm
GWO	Grey Wolf Optimization
I	Inclusivity
IQPSO	Improved Quantum behaved Particle Swarm Optimization
MR	Modification-Revealing test cases
PSO	Particle Swarm Optimization
QPSO	Quantum behaved Particle Swarm Optimization
SIR	Software Infrastructure Repository
TCP	Test Case Prioritization
TCR	Test Case Reduction
TCS	Test Case Selection
TFP	Test Fault Matrix
TRP	Test Reduction Percentage

### Nomenclature

$T_{i}$	test case of the test suite
PT	permuted test suite
f()	fitness function
*R*	requirement Set
$x_{i}$	position of a particle
$v_{i}$	velocity of a particle
$P_{i}$	personal best particles
*G*	global best particles
*w*	inertia weight
$c_{1},c_{2}$	social and cognitive components
*a*	local attractor
*u*	uniform random number
θ	contraction-expansion coefficient
Mbest	mean best of personal best particles
ϕ	random number
Maxit	,aximum number of iterations
Gus(x)	Gaussian distribution of x
α	evolution velocity coefficient
Gfit()	fitness value of global best particle
Pot(s)	potential of statement
*s*	statement covered by test case
P()	priority of test case
ST	selected Test Suite
RT	reduced Test Suite
max	maximum capacity of test suite to be selected
RSInd	indices of reduced array
Pop	maximum number of population
$p_{cr}$	crossover probability
$p_{m}$	mutation probability
*A*	loudness of bat
$r_{o}$	pulse emission rate of bat
$f_{min},f_{max}$	minimum and maximum frequency of bats
*n*	number of test cases
*m*	number of faults
st	selected test cases
emr	extracted modification-revealing test cases
totmr	total modification-revealing test cases
nfl	number of faults not covered
tfc	total faults covered
rcost	reduced cost of test suite
tcost	total cost of test suite
p,q	potential coefficients
